# MeCP2 Deficiency Alters the Response Selectivity of Prefrontal Cortical Neurons to Different Social Stimuli

**DOI:** 10.1523/ENEURO.0003-24.2024

**Published:** 2024-09-24

**Authors:** Natalie Boyle, Yipeng Li, Xiaoqian Sun, Pan Xu, Chien-Hsien Lai, Sarah Betts, Dian Guo, Rahul Simha, Chen Zeng, Jianyang Du, Hui Lu

**Affiliations:** ^1^Department of Pharmacology and Physiology, School of Medicine and Health Sciences, The George Washington University, Washington, DC 20037; ^2^Department of Computer Science, School of Engineering and Applied Science, The George Washington University, Washington, DC 20037; ^3^Department of Physics, Columbia College of Art and Sciences, The George Washington University, Washington, DC 20037; ^4^Department of Anatomy and Neurobiology, University of Tennessee Health Science Center, Memphis, Tennessee 38163; ^5^Neuroscience Institute, University of Tennessee Health Science Center, Memphis, Tennessee 38163

**Keywords:** in vivo calcium imaging, MeCP2, prefrontal cortex, prelimbic circuit, social discrimination, stimulus classification

## Abstract

Rett syndrome (RTT), a severe neurodevelopmental disorder caused by mutations in the MeCP2 gene, is characterized by cognitive and social deficits. Previous studies have noted hypoactivity in the medial prefrontal cortex (mPFC) pyramidal neurons of MeCP2-deficient mice (RTT mice) in response to both social and nonsocial stimuli. To further understand the neural mechanisms behind the social deficits of RTT mice, we monitored excitatory pyramidal neurons in the prelimbic region of the mPFC during social interactions in mice. These neurons’ activity was closely linked to social preference, especially in wild-type mice. However, RTT mice showed reduced social interest and corresponding hypoactivity in these neurons, indicating that impaired mPFC activity contributes to their social deficits. We identified six mPFC neural ensembles selectively tuned to various stimuli, with RTT mice recruiting fewer neurons to ensembles responsive to social interactions and consistently showing lower stimulus-ON ensemble transient rates. Despite these lower rates, RTT mice exhibited an increase in the percentage of social-ON neurons in later sessions, suggesting a compensatory mechanism for the decreased firing rate. This highlights the limited plasticity in the mPFC caused by MeCP2 deficiency and offers insights into the neural dynamics of social encoding. The presence of multifunctional neurons and those specifically responsive to social or object stimuli in the mPFC emphasizes its crucial role in complex behaviors and cognitive functions, with selective neuron engagement suggesting efficiency in neural activation that optimizes responses to environmental stimuli.

## Significance Statement

Unlike previous studies that have only shown altered prefrontal activity in response to stimuli in MeCP2-deficient models, our study identifies specific stimulus-tuned ensembles in the prefrontal cortex and their dysregulated activity patterns underlying social deficits in Rett syndrome (RTT). Utilizing innovative neural activity visualization tools, we revealed circuit-level evidence of the impairment caused by MeCP2 deficiency. The current research not only adds to the field of neurodevelopmental disorders and insights into the neurocircuitry of social behavior but also sheds light on the circuit-level mechanisms underlying a key behavioral phenotype of RTT.

## Introduction

Rett syndrome (RTT) is a rare neurodevelopmental disorder primarily affecting females, characterized by a range of cognitive and motor impairments as a result of mutations in the x-linked MeCP2 gene (Amir et al., 1999). Females with RTT develop normally until the first 6–18 months of life, after which they undergo a period of developmental regression and symptom onset (Hagberg et al., 1983). Impairments in social interaction and communication are among some of the hallmark symptoms of RTT, making it difficult for individuals with RTT to create meaningful social relationships (Schaevitz et al., 2010; Kaufmann et al., 2011). Social isolation and reduced social interaction have been associated with lower immune function and heightened neuroendocrine and cardiovascular activity, pointing toward a protective effect in the existence of such relationships (Seeman, 1996; Holt-Lunstad and Steptoe, 2022). It therefore remains crucial to investigate the neural correlates underlying this impairment in social interaction observed in RTT to prevent further damage caused by loss of MeCP2.

Social interaction remains a complex behavioral phenomenon, involving the use of sensory input, affective states, and various brain regions to elicit behavioral output in order to guide species survival (Ko, 2017; Chen and Hong, 2018; Xu et al., 2022). The medial prefrontal cortex (mPFC) acts as a hub to integrate social information from discrete modalities for top–down modulatory control of social behavior (Euston et al., 2012; Ko, 2017; Murugan et al., 2017; Huang et al., 2020). mPFC neural activity is found to be correlated with social approach behavior alongside social dominance rankings (Wang et al., 2011; Lee et al., 2016). Lesion and imaging studies in the mPFC have revealed patients with severe social impairments, while rodent studies indicate *E*/*I* imbalance within the mPFC can impair social interaction (Anderson et al., 1999; Forbes and Grafman, 2010; Yizhar et al., 2011; Sceniak et al., 2015).

Loss of MeCP2 causes both social and communication deficits in RTT individuals, emphasizing the necessity to investigate the intricate neural dynamics that underlie social interactions. MeCP2 loss has been found to impair preference for a novel conspecific in three-chamber tests of sociability and reduce interest and initiative in social interactions (Moretti et al., 2004; Garré et al., 2020). For example, in the tube test of social dominance, MeCP2 mutant mice consistently lost when faced with a wild-type (WT) mouse. When faced with another MeCP2 mutant, the same test increased in duration. This suggests the social impairments of MeCP2 mutant mice may be more apparent without a WT mouse to initiate the interaction (Moretti et al., 2004). In addition, previous work has shown that mPFC pyramidal neurons in MeCP2-deficient mice are hyporesponsive to both social and nonsocial stimuli (Xu et al., 2022). MeCP2 deficiencies therefore may restrict the circuit's ability to distinguish between different stimuli through dampening the responsiveness of excitatory circuits in the mPFC (Xu et al., 2022). However, what remains to be investigated is how this dampening of excitatory circuits affects ensemble recruitment and ensemble response selectivity to different stimuli.

In this study, we further delve into the circuit dynamics of social interaction in order to discern the neural ensembles responsive to specific stimuli interactions. Our findings reveal distinct ensembles selectively tuned to specific social interactions. Importantly, we highlight the unique activity patterns in RTT mice, particularly in altered recruitment and stability of neurons within ensembles across sessions. This work provides insights into how neural ensembles encode social information within the mPFC, utilizing a disease model of RTT to highlight differences in multifunctional neurons and ensemble activity in both healthy and disease states.

## Materials and Methods

### Experimental animals

Mouse maintenance and use were in accordance with NIH Guidelines and with the approval of the Institutional Animal Care and Use Committee of the George Washington University. *Mecp2*^+/−^ mice on the 129S6SvEvTac strain were obtained from the laboratory of Dr. Huda Zoghbi at Baylor College of Medicine, while Camk2-cre and PV-Cre mice of pure C57BL/6 background were purchased from Jackson Laboratory (JAX#005359). Female Camk2-cre, *Mecp2*^+/−^ (RTT) and Camk2-cre, *Mecp2*^+/+^ (WT) mice were obtained by breeding male Camk2-cre mice and female *Mecp2*^+/−^ mice, used for imaging excitatory neurons. We conducted experiments on WT and RTT mice of 3.5–4 months, following the same experimental procedure. Animals were given *ad libitum* access to standard mouse chow and water, housed four to five per cage in a temperature- (23 ± 1°C) and humidity (50 ± 10%)-controlled room with a 12 h light/dark cycle.

### Virus injection and GRIN lens implantation

For imaging excitatory neurons, AAV-Efla-Flex-GCaMP6m (Baylor College of Medicine) was stereotaxically injected as previously described (Belzung et al., 2014). Briefly, Camk2-cre mice (RTT and WT) were anesthetized and placed in a stereotaxic frame (Neurostar); then a 1.1-mm-diameter craniotomy [anterior–posterior (AP), +1.95 mm; medial–lateral (ML), −0.5 mm] was made with a high-speed rotary stereotaxic drill (Model 1474, AgnTho's AB). The virus was injected unilaterally (Nanoject II, Drummond Scientific) into the left region of the mouse prelimbic cortex, with the stereotaxic coordinates from the bregma: AP, +1.95 mm; ML, −0.35 mm; and dorsal–ventral (DV), −2.3 to −2.5 following a high-resolution atlas. A total of 600 nl of the virus (diluted with 600 nl PBS) was injected at the rate of 30 nl/min, and the needle was left in place for an additional 5 min after injection. Then, a 1-mm-diameter gradient-index (GRIN) lens (Inscopix) was lowered into the left prelimbic region (AP, +1.95 mm; ML, ±0.35 mm; DV, −2.1 to −2.3 mm), 0.2 mm above the virus injection site, at the speed of 50 µm/min, and then cemented in place (Metabond S380, Parkell). Mice were allowed to recover on a heating pad and thereafter closely monitored for 7 d, during which they received a daily injection of analgesic.

### Baseplate attachment

Three to four weeks after surgery, the virus expression in the anesthetized mouse was confirmed with a miniaturized microscope (Inscopix). If GCaMP^+^ neurons were visible and clear, the microscope attached to a baseplate would be hung above the mouse's skull window and lowered to assess the focus plane. Then, the baseplate was dental cemented onto the skull and capped with a cover, with the microscope unattached. Before the behavioral test, mice were habituated to the environment of the test room with a mounted dummy microscope and handled for 5–7 d, 40 min each day.

### Behavioral tests

Each day, only one behavioral test was conducted, and in each test the chamber was cleaned with 70% ethanol between trials. TopScan behavior analysis system (Clever Sys) was used to monitor the animal behaviors and would send a transistor-transistor logic (TTL) signal simultaneously to trigger the microscope recording neural activity at the beginning of each test.

#### Social approach test

This test was carried out according to the previous protocol (Lee et al., 2016) with some improvements. To facilitate in vivo imaging with the miniaturized microscope, the conventional three-chamber apparatus was modified to one open square box (45 × 10 × 20 cm) with two small removable lateral chambers (10 × 10 × 40 cm). The two end chambers were separated from the center open box by 1-cm-spaced thin metal wires to allow mice to interact with stimuli. Each test consisted of three 10 min sessions, which were conducted following a 10 min habituation period in the center open box. In Session 1 (S1), an age- and weight-matched strange same-sex conspecific (the first social stimulus, M1) and inanimate object (nonsocial stimulus, O) were separately placed in the two lateral end chambers randomly. In Session 2 (S2), the positions of those two stimuli were switched, which was designed to diminish the spatial influence in stimuli-induced neural activities. A mouse's preference to interact with a social stimulus rather than an inanimate object was used to evaluate their sociability. In Session 3 (S3), a second age- and weight-matched same–sex conspecific (new social stimulus, M2) was used to replace the object in the lateral end chamber. This session was used to evaluate social novelty preference, reflected by each mouse's propensity to spend more time with a new conspecific than with a familiar one. The time spent involved in a social interaction, object interaction, social zone, object zone, and middle zone were calculated.

### Calcium imaging with miniature microscope

Imaging in freely moving mice was performed using a head-mounted miniaturized microscope (nVista HD 2.0.4, Inscopix) triggered by a TTL pulse from the TopScan software to achieve simultaneous acquisitions of calcium signal and behavioral video. The microscope was mounted onto each mouse's head immediately prior to imaging. Calcium imaging data were acquired at a frame rate of 15 Hz and at 1,024 × 1,024 pixels. The LED power was set to 0.3–1 mW, and the gain was 1 to 2 depending on fluorescence intensity. Each individual mouse used the same imaging parameters of itself across three sessions.

### Data analysis

#### Behavior

Behavioral data were automatically tracked by top–down movies using the TopScan behavioral data acquisition software (Clever Sys). The 2D locations of mice were also tracked and defined as different zones, including social zone, object zone, and middle zone within the test chamber. The type and duration of detailed behavioral events throughout different test zones were recognized and extracted by the software, including social interaction with M1 (O), staying in social zone, grooming in the social zone, and approaching to M1 (O).

#### Calcium image processing

Calcium images were processed off-line using Inscopix Data Processing Software (version 1.2.1). Briefly, raw movies were processed through preprocessing, spatial filtering, and motion correction subsequently. For normalizing the calcium signal, the average projection of filtered movies was generated as the background fluorescence (*F*_0_), and instantaneous normalized Ca^2+^ fluorescent signals (Δ*F*/*F*) was calculated according to the formula, (Δ*F*/*F*)_i _= (*F*_i_ − *F*_0_) / *F*_0_, where i represents each frame. Then, individual cells were identified using the principal component and independent component analyses with no spatial or temporal downsampling, and the regions of interest were selected as candidate cells based on signal and image. Time-stamped traces of neurons were exported to Python (v3.0), where custom-written scripts were used for analysis onward. For calculating the calcium activity, spikes (i.e., transients) from fluorescence traces were predicted using unsupervised learning method sparse non-negative deconvolution (Pnevmatikakis and Giovannucci, 2017; Vogelstein et al., 2010) and near-online OASIS algorithm (Friedrich et al., 2017). To observe the neural activity involved in one specific behavior, we aligned the frames of calcium image data and frames of behavioral data with one another and marked the image frames with the corresponding behavioral event labels. The frequency of the transients, termed as “transition rate,” was quantified in transients per minute. This was achieved by aligning the imaging frames with the corresponding behavioral data, allowing us to count the number of calcium transients occurring while the mouse navigated through each of the five designated zones. The “amplitude” of these transients was measured as the change in fluorescence intensity (Δ*F*/*F*) relative to the baseline, using custom-written scripts in Python (version 3.8). This approach provides a standardized measure of the peak intensity of each calcium transient observed during the experimental sessions. In total, the calcium transients of 529 neurons from nine WT mice and 400 neurons from eight RTT mice were analyzed. The average number of neurons identified per mouse ranges from 37 to 108.

### Identification of interaction-tuned neural ensembles

To identify the interaction-tuned neural ensembles in each session, we evaluated the response preference of each neuron to one specific stimulus interaction. In brief, we first calculated the actual similarity (Sa) between vectors of calcium trace (ck) and behavior interaction (b), using the formula 2*b* *c*_k_ / (|*b*|^2 ^+ |*c*_k_|^2^) (Liang et al., 2018). Then, the behavior vector was randomly shuffled used to calculate a new similarity (Ss) with a neural trace for a neuron, which was repeated 5,000 times to generate Ss distribution histogram. The neuron was classified as ON if its Sa was greater than 99.95% of the Ss distribution; conversely, it was an OFF neuron if Sa was <0.05% of the Ss distribution.

Otherwise, it belonged to Others ensemble with Sa falling between 0.05 and 99.95%.

After the neuron classification, the proportion of ON, OFF, and Other neurons of each session were calculated for each mouse. Meanwhile, the transient rate and amplitude of those neuron ensembles were also calculated for each mouse in each session.

#### Neuron–behavior correlation analysis for neural ensemble

The correlation between calcium neural activity and specific behaviors within one neural ensemble was evaluated in two ways. Firstly, we calculated Pearson’s correlation coefficient between the averaged calcium activity of all neurons and behavior vector, which represents the coding ability of a neuron ensemble. Secondly, we calculated the average of individual Pearson’s correlation coefficients between each individual neural activity and behavior vector, which reflects the representing ability of all individual neurons within an ensemble.

#### Neural ensemble overlap percentage

For a pair of ensembles, such as *X* and *Y*, we first found the number of the overlapped neurons (*N*_XY_) between two ensembles. Meanwhile, the neuron number of ensemble *X* and *Y* were marked as *N*_X_ and *N*_Y_. Thus, the overlap percentage was calculated by the formula 2*N*_XY_ / (*N*_X _+ *N*_Y_). The range of overlap was from 0 to 1. For estimating the chance level of overlap, the probability of one neuron being randomly assigned into the ensemble *X* is *N*_X_ / *N* and into ensemble *Y* is *N*_Y_ / *N*. Assuming these two procedures are independent to each other, then the probability of one neuron falls into the overlap portion of ensemble *X*, and ensemble *Y* is *N*_X_ *N*_Y_ / *N*^2^. Consequently, the expected average number of randomly assigned neurons into *X* and *Y*, given whole population *N*, is *N*_X_ *N*_Y_ / *N*. To avoid any floats in the predicted value, we round the resulting numbers down to the nearest integers. The number of neurons identified for each category ranges from 20 to 88.

### Statistics

All statistical analyses were performed using SPSS Statistics (version 24, IBM), Excel (Microsoft), and Python custom scripts. Since all of the data passed the normal distribution test (D’Agostino and Pearson’s), a two-tailed paired sample or unpaired *t* test was applied for comparison. For multiple-factor comparison, two-way RM ANOVA was used, followed by Bonferroni’s corrected post hoc comparisons. Statistical significance was taken as *p < 0.05; **p < 0.01; and ***p < 0.001. All data are represented as mean ± SEM unless otherwise specified. The linear curve was fitted to indicate transient rate–behavior correlation, and the correlation was tested by regression analysis.

### Data and materials availability

All data and codes in the main text or the supplementary materials are available upon request.

## Results

### Distinct mPFC ensembles are selectively tuned to specific stimuli

**Figure 1.**
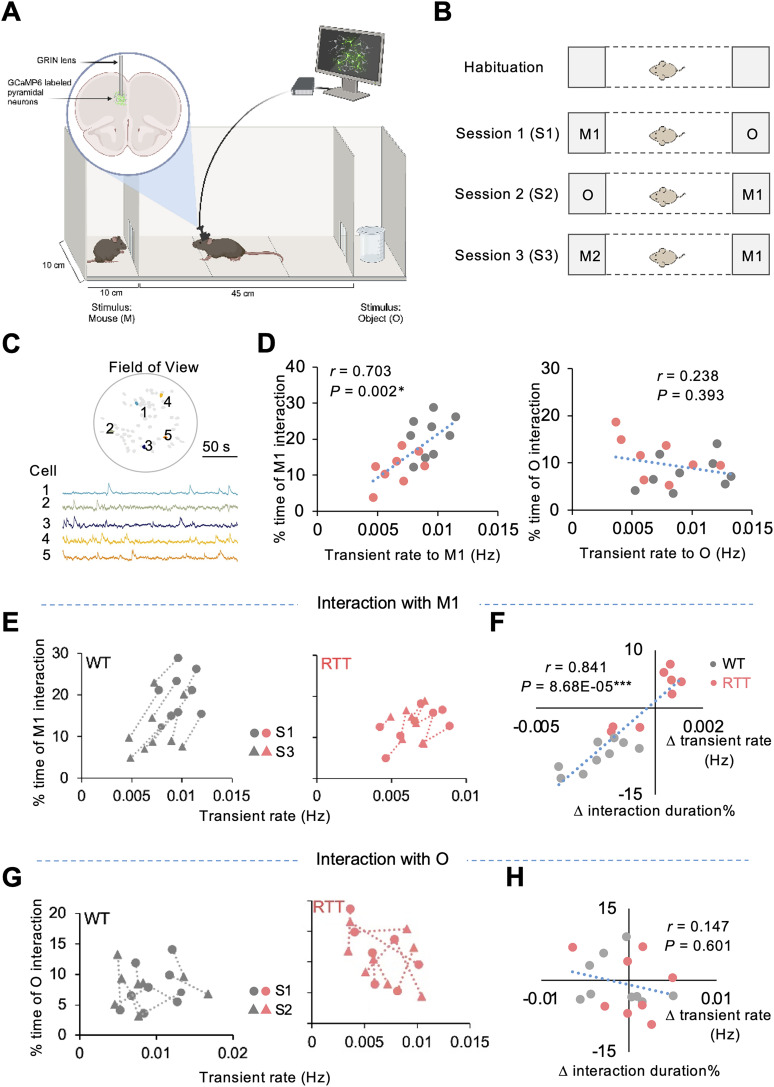
The reduced transient rate of mPFC neural circuit is related to the lack of interest in social interactions of RTT mice. ***A***, Schematic of the experimental approach. ***B***, Schematic of social interaction test. Habituation allows the animal to habituate in the middle chamber for 10 min with empty end chambers. In the first 10 min test session (S1), a strange mouse (M1) and an object (O) were placed in the end chambers; S2 used the same stimuli but swapped their positions; in S3 a new mouse (M2) replaced O, so that the subject mouse must choose whether to interact with a familiar or strange mouse (M1 vs M2). ***C***, Top, The field of view under a GRIN lens in one mouse with identified neurons numbered and colored. Bottom, Fluorescence traces of example neurons marked in the above panel. ***D***, Correlation between the transient rate and proportion of time spent interacting with a mouse (left) or object (right) in individual WT (*n* = 9) and RTT (*n* = 8) mice in S1. Each dot represents an individual mouse; Pearson's correlation coefficients were calculated across genotypes. *p < 0.05, regression. ***E***, Relationship between the transient rate and amount of time that WT and RTT mice respond to M1 in S1 and S3. Circles and triangles represent individual mice in S1 versus S3; dashed lines connect values from the same mouse over two different sessions. ***F***, Relationship between the changes of the transient rate and the duration of interaction of WT and RTT mice with M1 over S1 to S3. Each dot represents an individual mouse. Pearson's correlation coefficients were calculated across genotypes. ***p < 0.001, regression. ***G*** and ***H*** are similar to ***E*** and ***F***, respectively, except in response to O in the first two sessions.

In order to identify neural ensembles involved in social encoding, we monitored the activity of excitatory pyramidal neurons in the prelimbic region of the mPFC using head-mounted miniature microscopes during a three-chamber social test (Fig. 1*A*). Habituation to the apparatus was followed by three sessions of social interaction, with varying stimuli placed in the end chambers. In S1, the experimental mouse can interact with either a conspecific (M1) or an object (O). In S2, the position of M1 and O are switched in the apparatus. In S3, the object is replaced with a novel mouse (M2; Fig. 1*B*). We identified 60–120 excitatory pyramidal neurons expressing the Ca^2+^ indicator, GCaMP6m, from each mouse, without bleaching effects in recording (Fig. 1*C*). We found that the average Ca^2+^ transient rate of the mPFC ensemble for both WT and RTT mice correlated more with the amount of time they spent near another mouse (Fig. 1*D*, left) than with time spent near the object (Fig. 1*D*, right). Notably, RTT mice displayed reduced social interest, and this was accompanied by hypoactivity in their mPFC pyramidal neurons, as indicated by the lower positioning of the data point distribution from the RTT group (Fig. 1*D*, left). As the behavioral and neural responses to interaction with the same stimulus changed across different sessions, we wondered whether the observed neural activity–behavior correlation would persist. For M1 interactions, the cross-session correlation between neural activity and interaction duration with M1 moved in opposite directions for the WT and RTT groups (Fig. 1*E*,*F*). There was no clear trend for the cross-session differences between neural activity and interaction duration with O for any individual mouse (Fig. 1*G*,*H*). These results suggest that the activity level of the mPFC pyramidal neurons correlates with the mouse's social preference. Consequently, the hypoactivity of the mPFC excitatory ensemble contributes to the social deficits observed in RTT mice by impairing their ability to distinguish between social and nonsocial stimuli (Xu et al., 2022).

**Figure 2.**
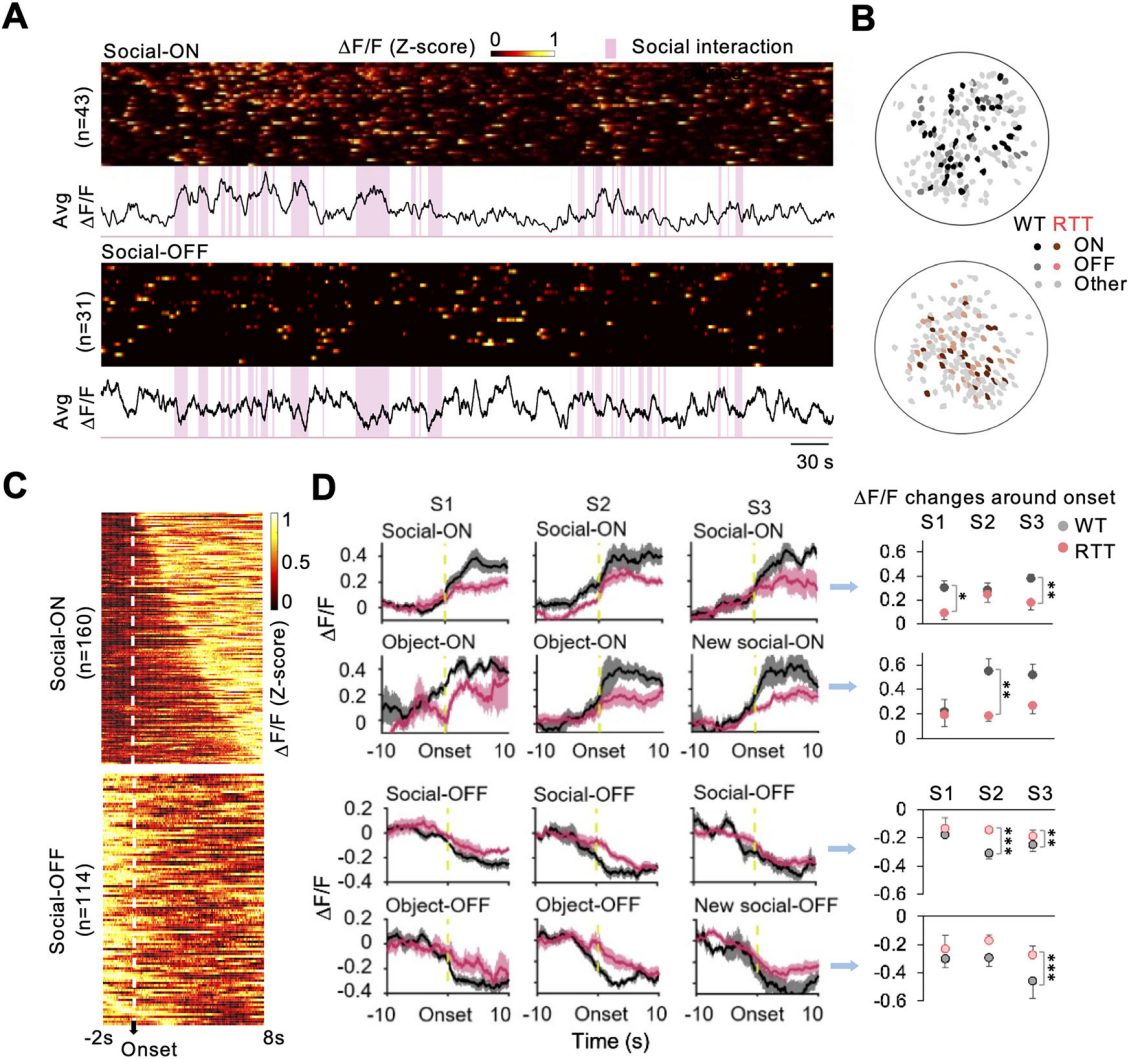
Neurons in the mPFC respond to different stimuli. ***A***, Raster plots of individual neural activities and calcium traces of averaged group activity of social-ON and social-OFF neurons from one representative mouse in S1. Vertical pink bars indicate discrete episodes of social interaction. ***B***, Spatial distributions of social-ON, social-OFF, and other neurons from one WT and one RTT mouse at S1. ***C***, Raster plots of calcium activities of individual social-ON and social-OFF neurons around the onset of social interaction (2 s before and 8 s after), sorted by the time points of maximal (ON neurons) or minimal activities (OFF neurons) appearance. ***D***, Left, Averaged calcium traces of different stimulus-tuned ON/OFF neural ensembles around the onset of the corresponding interactions (10 s before to 10 s after). Solid lines and shaded regions represent the averaged value and SEM, respectively. Right, Amplitude changes of ON and OFF ensembles (as shown in left traces) around the onset of the interactions with the stimuli they were tuned to (10 s after minus 10 s before). Δ*F*/*F*, change in fluorescence over baseline fluorescence intensity. Values are represented as mean ± SEM. *p < 0.05; **p < 0.01; ***p < 0.001; RTT (*n* = 8) mice versus WT (*n* = 9), two-way RM ANOVA with Bonferroni-corrected post hoc comparisons.

To verify that the neurons in the prelimbic region are involved in the representation of (social and nonsocial) stimuli, we identified neurons that respond preferentially to interaction with a specific stimulus by calculating the neuron's activity at the start of each behavior and then comparing this activity level with the neuron's own chance level of activity (Hamm et al., 2017; Liang et al., 2018). Neurons whose activity strongly correlated with the start of a particular behavior were classified as part of an ensemble active for a specific interaction (ON neurons), while those whose activity level anticorrelated with the behavior were classified as OFF neurons (Fig. 2*A*). We thus identified six ensembles that were selectively tuned (either ON or OFF) to a social, object, or new social stimulus, respectively. As a whole, the ensembles reliably and effectively encoded specific stimuli by compensating for temporal variation in the activity of individual neurons. ON and OFF neurons were scattered in the field of view (Fig. 2*B*) and increased or decreased in activity around the onset of the interaction with a specific stimulus in both genotypes (Fig. 2*C*,*D*). RTT ensembles, however, especially the social-ON and new social-ON ensembles, consistently showed a lower amplitude of response (Fig. 2*D*).

### Altered transient rates in MeCP2-deficient mPFC ensembles reveal impaired information coding

**Figure 3.**
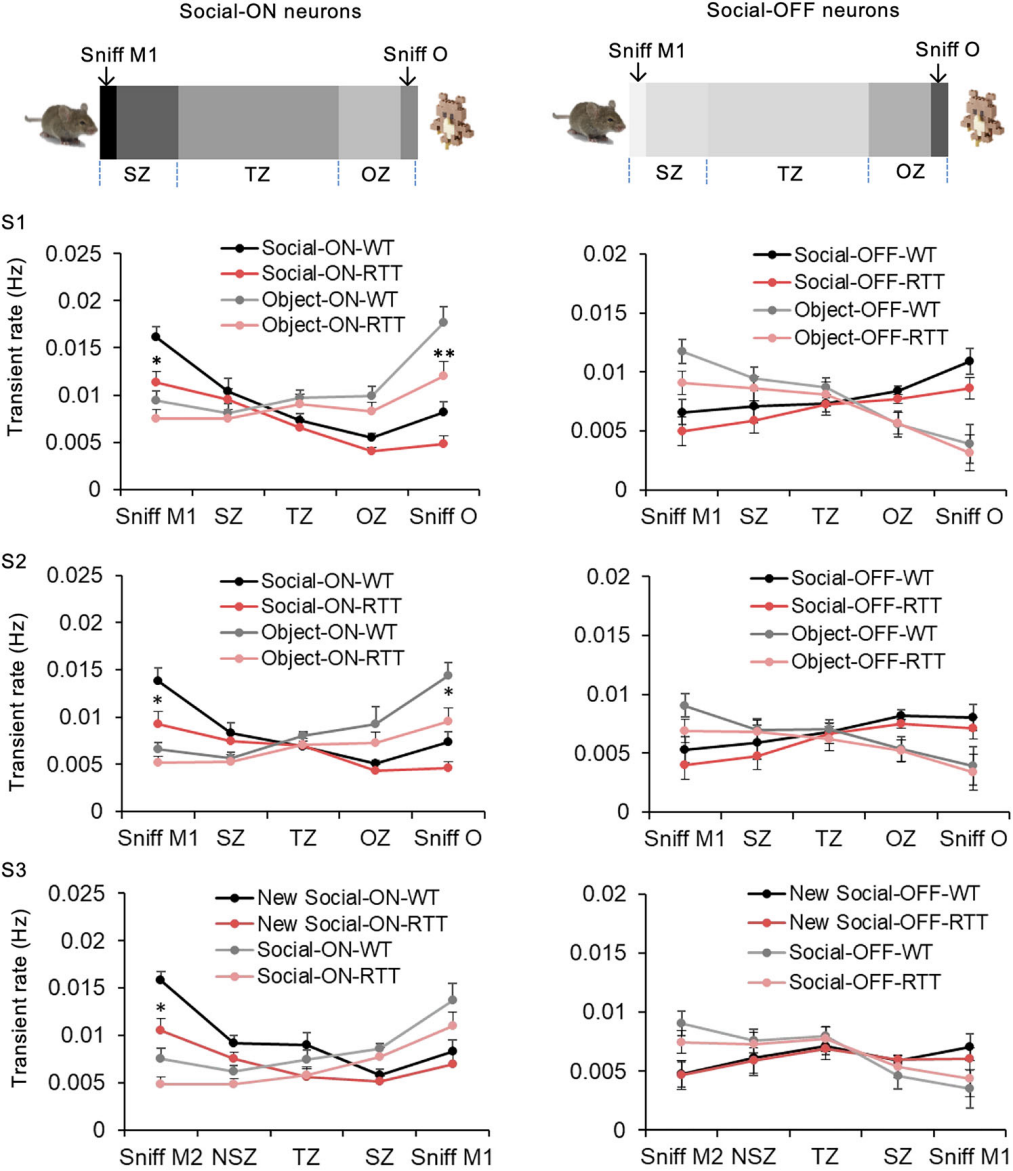
Transient rates of mPFC neural ensembles across different zones did not change as much in RTT mice as in WT mice. Top, Diagrams showing the gradient of responses of social-ON and social-OFF ensembles by spatial field from one representative mouse; darker gray means higher response. Bottom, The transient rate of stimulus-tuned ON/OFF ensembles in WT mice (black, gray) and RTT mice (red, pink) across different locations of the chamber. “Sniff” means the mouse was at the end of the chamber and interacting with a stimulus; social zone (SZ) and object zone (OZ) indicate the 10 cm regions of the central chamber that are nearest the end chambers which are M1 and O, respectively; transition zone (TZ) indicates the middle chamber. Data are represented as mean ± SEM. *p < 0.05; RTT (*n* = 8) mice versus WT (*n* = 9); two-way RM ANOVA with Bonferroni-corrected post hoc comparisons.

The response selectivity of the ensemble could be represented by activity changes in response to different stimuli. The ON/OFF ensembles always showed their highest/lowest value in the vicinity of the stimuli they represented, for both WT and RTT mice (Fig. 3). The transient rates of ON and OFF ensembles changed as mice moved into other areas of the three-chamber apparatus. Overall, however, this response selectivity to specific stimuli was sharper in WT than in RTT mice. When we considered only the immediate vicinity of one stimulus (where interactive sniffing happened), the corresponding stimulus-tuned ON ensemble displayed a much lower transient rate in RTT mice than WT, whereas the OFF ensembles did not differ between the two groups (Fig. 3, right).

### The proportion of neurons recruited to distinct ensembles differed between WT and RTT mice

**Figure 4.**
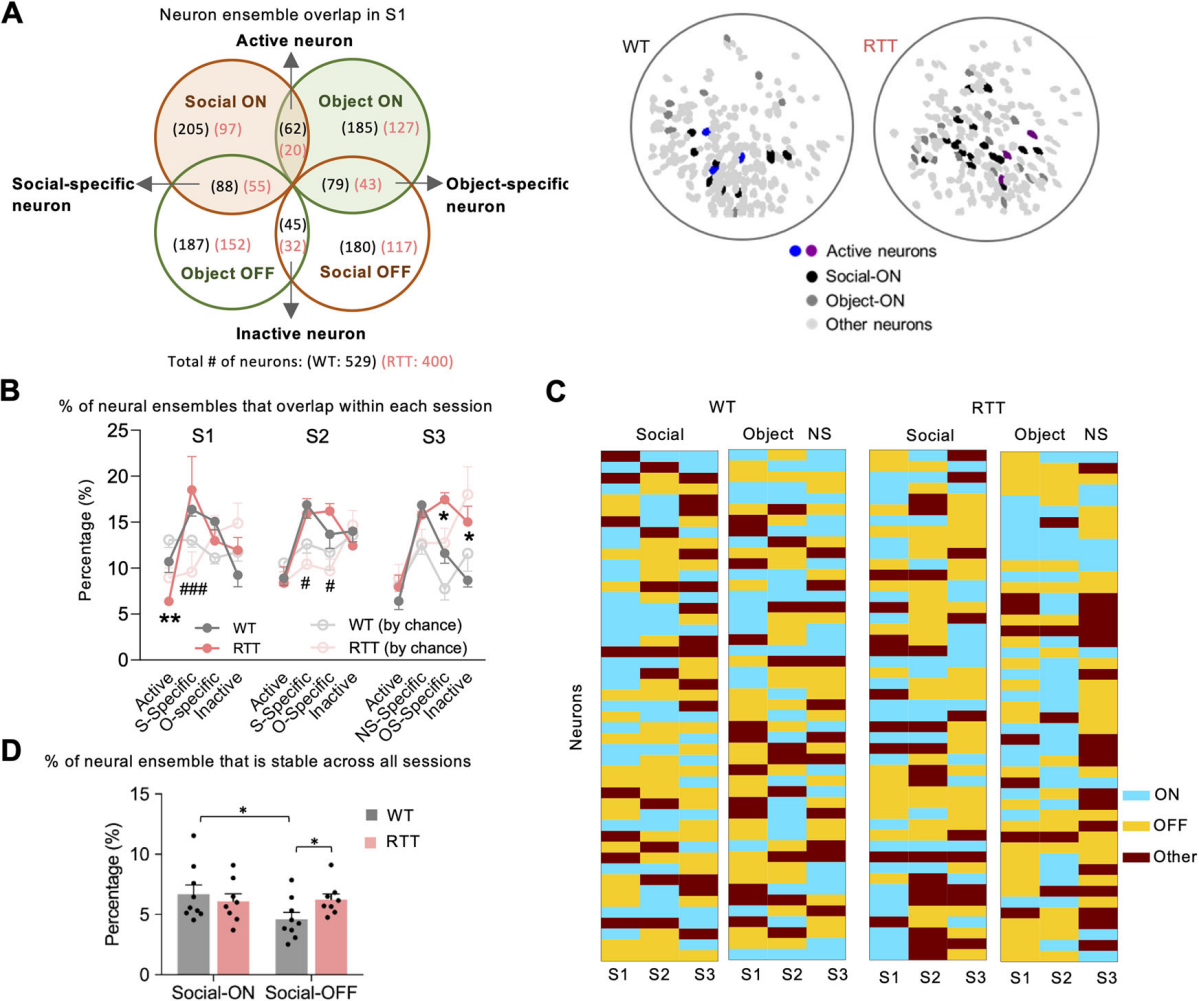
Neural recruitment to ensembles is altered in RTT mice. ***A***, Left, Four groups of overlapped neurons, categorized by how they respond to two different stimuli within one session (here is S1). Numbers in parentheses represent the number of neurons in the category. Black, WT; pink, RTT. Right, Spatial distribution of the active neurons of WT (blue) and RTT (purple) mice. ***B***, Proportion of the neural ensembles (as defined in ***A***) in each session of WT and RTT mice. S, social; O, object; NS, new social (novel mouse); OS, old social (familiar mouse). The neurons that are most stable in WT mice are always specific to (new) social interaction, whereas those in the RTT mice are specific to the object in S2 and to the familiar mouse in S3. Data are shown as mean ± SEM. *p < 0.05; **p < 0.01; two-way RM ANOVA with Bonferroni-corrected post hoc comparisons. ***C***, The map of ensemble coding properties across three testing sessions from a representative WT and RTT mouse. ***D***, The percentage of neurons that were always tuned to M1 (either ON or OFF) over all three sessions. Data are shown as mean ± SEM. *p < 0.05; RTT (*n* = 8) versus WT (*n* = 9); two-tailed Student's *t* test

Neural ensembles recruit neurons to encode complex information, so it is not surprising that the ensemble is not homogeneous (Antic et al., 2018). In both WT and RTT mice, we identified six distinct ensembles in the mPFC that reliably encode for different stimuli (Fig. 4). Each ensemble is selectively tuned (ON or OFF) to either a social, object, or new social stimulus. Transient rates of individual ON or OFF ensembles displayed their highest or lowest (respectively) rates when in the vicinity of the stimuli in which they encoded. Some neurons were recruited to multiple ensembles to participate in encoding more than one type of information (Fig. 4*A*). We calculated the percentage of these multifunctional neurons and observed that the proportion of active and inactive neurons tended to be lower than expected by chance. Conversely, the percentages of social- or object-specific neurons were significantly higher than the chance level (Fig. 4*B*), indicating that certain neurons in the mPFC are specialized or primarily active in response to social- or object-related stimuli. This specialization suggests that these neurons play a distinct role in processing these types of stimuli. Furthermore, we found that RTT mice had fewer active neurons, which were tuned to encode both social and nonsocial information, in S1, indicating impairment of information coding in the RTT mPFC circuit. However, in S3, RTT mice recruited more neurons that responded specifically to the familiar (“old”) social stimuli and more inactive neurons that were tuned OFF to both old and new social stimuli (Fig. 4*B*).

In different sessions, we also noticed that this recruitment process was very dynamic (Fig. 4*C*). The percentage of ON or OFF neurons that remained tuned to M1 across all three sessions was very low (below 8%), but only the WT ensemble showed a difference between the proportions of stable ON and OFF neurons (Fig. 4*D*). Consistent with previous finding (Xu et al., 2022), the RTT circuit is less flexible, reflected by recruiting fewer neurons to the ensemble for social interaction, and less dynamic (more neurons remain stable) in responding specifically to social stimuli.

## Discussion

We have identified distinct ensembles in the mPFC that reliably encode for different stimuli. Each ensemble is selectively tuned (ON or OFF) to either a social, object, or new social stimulus. Transient rates of individual ON or OFF ensembles displayed their highest or lowest (respectively) rates when in the vicinity of the stimuli in which they encoded. Additionally, some neurons were recruited to multiple ensembles, implying they are able to encode for more than one type of information. This pattern of encoding has been previously reported within the mPFC, further providing evidence that the mPFC is able to form rich contextual representations through sensory cues and actions and carry information regarding social salience (Hyman et al., 2012; Liang et al., 2018). The presence of multifunctional neurons (those that respond to more than one type of stimulus) and neurons specifically responsive to social or object stimuli suggests functional diversity within the mPFC. This diversity is crucial for complex behaviors and cognitive functions that involve this brain region. The observation that the percentages of active and inactive neurons tended to be lower than expected by chance (Fig. 3*B*) could indicate that a significant portion of the mPFC is selectively engaged by specific stimuli rather than being generally active. This could reflect an efficiency in neural activation, where neurons are conserved for specific, rather than general, activations.

Our work has additionally shown the existence of alterations in mPFC neural ensemble dynamics in MeCP2-deficient mice during social interactions. RTT ON ensembles consistently displayed lower transient rates in the immediate vicinity of stimuli alongside a lower amplitude of response. The observed decrease in ON ensemble transient rate and amplitude occurred regardless of interaction with a mouse or an object. This coincides with previous research indicating a deficit in pattern decorrelation in RTT mice through hypoactivity in mPFC pyramidal neurons (Xu et al., 2022). With the ability to distinguish stimuli impaired, RTT mice may be less likely to engage in social interaction or seek social interaction over object interaction.

In a similar sense, we observed the social-ON ensemble in RTT mice to show a strong selectivity to the familiar mouse in S3. In addition to impairments in stimuli discrimination, this supports previous evidence that there exists a lack of preference for social novelty in RTT mice (Schaevitz et al., 2010). Social novelty seeking is an innate behavior in rodents, with distinct ON and OFF ensembles found to be tuned to carry information on social salience and novelty (Liang et al., 2018). Notably, neurons in the mPFC are capable of rapidly forming ensemble codes for novel stimulus associations, suggesting an impairment of this capability in RTT mice (Takehara-Nishiuchi et al., 2020). In addition to pyramidal activity, previous studies exploring social novelty in RTT models suggest that GABAergic interneurons in the mPFC may contribute to impaired stimuli discrimination. RTT parvalbumin neurons in the mPFC display higher activity levels than those in WT mice during stimuli interactions, albeit with limited dynamic range (Xu et al., 2022). Moreover, novelty-cue preferred interneurons are found to respond nonpreferentially in RTT mice, indicting social novelty preference may further be regulated through interneuron hyperactivity (Zhao et al., 2022).

RTT mice displayed impairments in mPFC neural ensemble recruitment, as we observed fewer neurons responding to social interaction. Of particular interest is the diminished recruitment of neurons encoding both social and nonsocial information in RTT mice compared with WT, suggesting an impairment in information coding. Because MeCP2 loss universally impacts all brain regions, this impairment in ensemble recruitment continues to exist outside of the mPFC. RTT mice have impairments in hippocampal ensemble recruitment, which is found to disrupt long-term contextual memory recall (He et al., 2022). Thus, RTT impairments in information coding due to impaired ensemble recruitment span across regions and impact various aspects of social behavior, cognition, and stimulus processing.

Despite impairments in social discrimination and novelty seeking, our results also highlight the limited plasticity that remains intact in the RTT mPFC. RTT mice were found to display an increase in the percentage of social-ON neurons in later sessions—a potential compensatory mechanism for the low firing rate. This adaptive response suggests that despite the altered circuitry, RTT circuits retain enough plasticity to counterbalance deficits in firing rates. The idea that RTT circuits maintain some form of plasticity has additionally been noted to occur in other regions of the brain. In the motor cortical circuit of MeCP2-deficient mice, mice are found to retain sufficient plasticity to support some motor learning (Yue et al., 2021). Furthermore, research has found that presymptomatic training dramatically improves the performance of specific motor and memory tasks (Achilly et al., 2021). In a broader context, these findings highlight the resilience of neural circuits in RTT. Despite the biological disruptions caused by lack of MeCP2, adaptive mechanisms continue to exist and may contribute to the preservation of essential neural functions. These findings indicate that there are ways to manipulate the RTT circuitry to induce learning and memory and that MeCP2-deficient neuronal circuitry is intact enough to retain a limited form of plasticity. This resilience offers promising avenues for future therapeutic targets aimed at harnessing and enhancing these adaptive mechanisms to preserve ensemble functions.

## Synthesis

Reviewing Editor: Julie Bakker, University of Liege Decisions are customarily a result of the Reviewing Editor and the peer reviewers coming together and discussing their recommendations until a consensus is reached. When revisions are invited, a fact-based synthesis statement explaining their decision and outlining what is needed to prepare a revision will be listed below. The following reviewer(s) agreed to reveal their identity: Eunee Lee. Note: If this manuscript was transferred from JNeurosci and a decision was made to accept the manuscript without peer review, a brief statement to this effect will instead be what is listed below.

Two reviewers have seen the ms and made comments, please pay close attention to them.

Reviewer 1:

1.To enhance the interpretive value of the overlap analysis, it would be beneficial to provide a comparison against the degree of overlap expected by chance. Specifically, the designation of on or off cells as the top or bottom 0.5% of the total verified neuron population is noted, with an identification of 60-120 neurons per experiment. This suggests an estimate of approximately 5-6 neurons per group as on or off cells. However, presenting only the proportion of overlap could potentially be misleading to readers, as the actual numbers of neurons with overlap or ensemble stability are less than 5-6. For greater transparency and to bolster the data's credibility, the raw data should be presented as the number of neurons calculated for each proportion. If the number of neurons in each group is minimal, the statistical robustness and the data's reliability may be called into question.

Furthermore, Figures 4 and 5, which present non-overlapped and overlapped proportions respectively, seem to conduct redundant analyses. In particular, Figure 4 exhibits a complex calculation that is challenging to comprehend without a detailed explanation. Additionally, the statistical methodology employed is neither clear nor convincingly justified. Considering the redundancy in the message of Figures 4 and 5, it is recommended that Figure 4 be omitted to facilitate a clearer presentation of the data.

2.Figures 2 and 3 appear to be derived from the same dataset; however, the methods section or main text lacks a clear definition of the terms 'transition rate' and 'amplitude'. Given the various methods to define the main peak of a calcium transient, it is crucial that the authors provide an explicit description of how these terms are defined and measured in their study.

3.The manuscript states in lines 281-282 that more attractive stimuli elicited greater neuronal amplitudes. The use of the term "attractiveness" in this context is problematic, as it anthropomorphizes neuronal responses. It is imperative that the manuscript maintain a scientific and objective perspective. The authors should reconsider this wording and describe the neuronal activity in terms that accurately reflect the observed phenomena without inferring subjective experiences of the neurons.

4.The significant statement includes a statement regarding 'slight plasticity retained by the MeCP2-deficient circuit,' inferred from data indicating that the number of overlaps from the RTT exceeds those in WT type mice. However, this conclusion may be an overinterpretation of the data. The presence of a greater number of overlaps in RTT mice compared to WT does not necessarily equate to slight plasticity. The authors should revise this statement to more accurately reflect the findings without overextending the interpretation.

5.Regarding the phrase found on page 2, line 33, the term "nonsupprative" is unclear. If this is a typographical error, it should be corrected. If it is a term intended to convey a specific meaning, further clarification is needed to ensure that readers understand the concept being communicated.

6. The author should present the behavior data during calcium imaging analysis. The total duration and trial number would be better to be shown in the comparison of WT and RTT mice. and the total number of neurons and the average number of neurons identified per mouse should be described for transparency of the experiment.

Reviewer 2:

The authors employed a miniaturized in-vivo fluorescence microscope to observe the activity of discrete mPFC neural ensembles in female wild-type and MeCP2-deficient mice during the 3-Chamber test of sociability. They identified six mPFC neural ensembles that were selectively tuned to different stimuli, and found that RTT mice recruited fewer neurons to ensembles responding to social interaction and consistently produced lower stimulus-ON ensemble transient rates.

Concern #1: Lack of behavior data. there is no behavior data for RTT mice and WT mice. Does the behavior abnormality correlate with the PFC abnormal ensembles?

Concern #2: Lack of explanation/interoperation for the random de-activation nature of X chromosomes. Neurons in the mPFC are a mix of MeCP2 intact and deficient neurons.

## Author Response

The reviewers all provided very constructive comments, which we have been happy to address. For the sake of clarity, we present the reviewers' comments in italics and our responses in regular font. In-text citations are listed in full, alphabetically, at the end of this document.

Reviewer #1 1.To enhance the interpretive value of the overlap analysis, it would be beneficial to provide a comparison against the degree of overlap expected by chance. Specifically, the designation of on or off cells as the top or bottom 0.5% of the total verified neuron population is noted, with an identification of 60-120 neurons per experiment. This suggests an estimate of approximately 5-6 neurons per group as on or off cells. However, presenting only the proportion of overlap could potentially be misleading to readers, as the actual numbers of neurons with overlap or ensemble stability are less than 5-6. For greater transparency and to bolster the data's credibility, the raw data should be presented as the number of neurons calculated for each proportion. If the number of neurons in each group is minimal, the statistical robustness and the data's reliability may be called into question.

Thank you for your feedback regarding the classification of "on" and "off" neurons in our study. It seems there may be a misunderstanding concerning the methodology used to determine these categories. Specifically, the designation of "on" or "off" cells as the top or bottom 0.5% of the total verified neuron population does not represent a fixed number of neurons. Instead, this classification is based on their ranking according to real calcium-behavior similarity within a distribution generated through 5000 iterations of shuffling. To elaborate, each neuron's real calcium-behavior similarity is assessed against a distribution of calcium-behavior similarities derived from 5000 shuffled datasets. A neuron is categorized as an "on" neuron if its calcium-behavior similarity ranks in the top 0.5% of this distribution, and as an "off" neuron if it falls within the bottom 0.5%. Consequently, every neuron has the potential to be classified as an "on" neuron based on its performance relative to the shuffled baseline. This method is adopted from Liang et al., 2018 (Neuron, 100, 1-15). For further details, please refer to Figure S2B in this article, which illustrates the process of comparing calcium-behavior similarity for individual neurons. This approach ensures that our classification is robust, reflecting significant deviations from what would be expected by chance and providing a reliable measure of neuron behavior in response to experimental conditions. Additional details about the data analysis and the number of neurons analyzed are provided in the methods section. Briefly, we analyzed a total of 529 neurons from 9 wild-type (WT) mice and 400 neurons from 8 Rett Syndrome (RTT) mice. The raw number of neurons in each category for session 1 is presented in Fig. 3A. Tthe degree of overlap expected by chance for each category is plotted in light color in Fig. 3B. We edited the result section and added a discussion on this result (highlighted in yellow in the revised manuscript).

Here is how the chance level is calculated. The number of neurons that belong to both ensemble A and B by chance is defined as: ¬N_(A∩B)^C= (N_A N_B)/N Where N_i is the number of neurons in ensemble i and i∈{A,B} To be specific, the probability of one neuron being randomly assigned into the ensemble A is N_A/N and into ensemble B is N_B/N. Assuming these two procedures are independent to each other, then the probability of one neuron falls into the overlap portion of ensemble A and ensemble B is N_A N_B/N^2. Consequently, the expected average number of randomly assigned neurons into A and B, given whole population N, is N_A N_B/N. To avoid any floats in the predicted value, we round the resulting numbers down to the nearest integers. This information is added to the method section.

Furthermore, Figures 4 and 5, which present non-overlapped and overlapped proportions respectively, seem to conduct redundant analyses. In particular, Figure 4 exhibits a complex calculation that is challenging to comprehend without a detailed explanation. Additionally, the statistical methodology employed is neither clear nor convincingly justified. Considering the redundancy in the message of Figures 4 and 5, it is recommended that Figure 4 be omitted to facilitate a clearer presentation of the data.

We agree with the reviewer that Figure 4 may seem redundant. Accordingly, we have excluded it in the revised manuscript.

2. Figures 2 and 3 appear to be derived from the same dataset; however, the methods section or main text lacks a clear definition of the terms 'transition rate' and 'amplitude'. Given the various methods to define the main peak of a calcium transient, it is crucial that the authors provide an explicit description of how these terms are defined and measured in their study.

Thank you for your inquiry regarding the definitions of 'transition rate' and 'amplitude' as used in our study, particularly as presented in Figures 2 and 3.

In our analysis, calcium transients were identified from time-stamped neuronal traces using a peak-finding algorithm tailored for each cell. The frequency of these transients, termed as 'transition rate,' was quantified in transients per minute. This was achieved by aligning the imaging frames with the corresponding behavioral data, allowing us to count the number of calcium transients occurring while the mouse navigated through each of the five designated zones.

The 'amplitude' of these transients was measured as the change in fluorescence intensity (∆F/F) relative to the baseline, using custom-written scripts in Python (version 3.8). This approach provides a standardized measure of the peak intensity of each calcium transient observed during the experimental sessions.

We recognize the importance of clearly defining these terms and have added detailed descriptions of these methodologies in the revised methods section to ensure clarity and reproducibility of our findings.

3.The manuscript states in lines 281-282 that more attractive stimuli elicited greater neuronal amplitudes. The use of the term "attractiveness" in this context is problematic, as it anthropomorphizes neuronal responses. It is imperative that the manuscript maintain a scientific and objective perspective. The authors should reconsider this wording and describe the neuronal activity in terms that accurately reflect the observed phenomena without inferring subjective experiences of the neurons.

Thank you for pointing this out. We use "preferred" to replace "attractive".

4.The significant statement includes a statement regarding 'slight plasticity retained by the MeCP2-deficient circuit,' inferred from data indicating that the number of overlaps from the RTT exceeds those in WT type mice. However, this conclusion may be an overinterpretation of the data. The presence of a greater number of overlaps in RTT mice compared to WT does not necessarily equate to slight plasticity. The authors should revise this statement to more accurately reflect the findings without overextending the interpretation.

We have revised the significance to state, "..., we reveal circuit-level evidence of the impairment caused by MeCP2-deficiency." 5.Regarding the phrase found on page 2, line 33, the term "nonsupprative" is unclear. If this is a typographical error, it should be corrected. If it is a term intended to convey a specific meaning, further clarification is needed to ensure that readers understand the concept being communicated.

Thank you for catching this error. We have revised "nonsuppurative social interaction" to "reduced social interaction".

6. The author should present the behavior data during calcium imaging analysis. The total duration and trial number would be better to be shown in the comparison of WT and RTT mice. and the total number of neurons and the average number of neurons identified per mouse should be described for transparency of the experiment.

We have updated Figure 1 to include both the behavioral data for each mouse and the correlation between the total duration of social interaction and the activity of mPFC neurons for each individual mouse. Since each mouse will undergo the social test only once, the number of trials is equal to 1. Additionally, the total number of neurons and the average number of neurons identified per mouse are detailed in the methods section: "In total, the calcium transients of 529 neurons from 9 WT mice and 400 neurons from 8 RTT mice were analyzed. The average number of neurons identified per mouse ranges from 37 to 108." Reviewer #2 The authors employed a miniaturized in-vivo fluorescence microscope to observe the activity of discrete mPFC neural ensembles in female wild-type and MeCP2-deficient mice during the 3-Chamber test of sociability. They identified six mPFC neural ensembles that were selectively tuned to different stimuli, and found that RTT mice recruited fewer neurons to ensembles responding to social interaction and consistently produced lower stimulus-ON ensemble transient rates.

Concern #1: Lack of behavior data. there is no behavior data for RTT mice and WT mice. Does the behavior abnormality correlate with the PFC abnormal ensembles? We have updated Figure 1 to include both the behavioral data for each mouse and the correlation between the total duration of social interaction and the activity of mPFC neurons for each individual mouse. The significant correlation between these two parameters strongly suggests that the reduced duration of social interaction observed in RTT mice is closely associated with the hypoactivity of their mPFC pyramidal neurons.

Concern #2: Lack of explanation/interoperation for the random de-activation nature of X chromosomes. Neurons in the mPFC are a mix of MeCP2 intact and deficient neurons.

We are aware that the synaptic properties of MeCP2+ and MeCP2- neurons will differ, and that some effects of MeCP2 have been shown to be cell-autonomous while others are cell-non-autonomous (e.g. Maezawa et al, J. Neurosci., 2009, Asgarihafshejani et al, Neuroscience, 2019; Rakela et al, eLife, 2018).

We took two approaches to address the reviewer's concern. First, we thoroughly examined all of our data looking for a bimodal distribution of effects across cells, as would be expected if MeCP2-lacking and MeCP2-expressing cells show differences with respect to firing rates, decorrelation magnitude, or other measures. We found no evidence of bimodality.

Second, we looked into the literature. Achilly et al. (Nature 2021) recently looked at the effects of exercise in female RTT mice and examined hippocampal neurons. Both MeCP2+ and MeCP2- hippocampal cells were recruited during the Morris Water Maze task; as long as the MeCP2- cells were activated on that specific task, they showed enhanced dendritic arborization, as did MeCP2+ cells.

Third, compelling evidence from our previous work (Xu et al., Nature Communications, 2022) demonstrates that stimulating mPFC neurons in heterozygous female MeCP2-deficient mice, irrespective of their genotype, successfully restored social preferences when mPFC circuit dysfunction was rescued. This finding decisively underscores the close connection between behavioral abnormalities and circuit-level dysfunctions, rather than cellular-level genotypes, highlighting the critical role of neural circuits in governing behavior.

Moreover, in previous work (Lu et al., Neuron, 2016) we showed that three different MeCP2-related models (male Mecp2-null, female Mecp2-het, and Mecp2-overexpressing mice) all showed the very same phenotype at the circuit level, namely, hypersynchrony, impaired response to perturbations of E/I homeostasis, and impaired excitatory synaptic response in inhibitory neurons. Differences at the molecular or cellular level therefore do not necessarily manifest as differences at the circuit (or behavioral) level-otherwise we would not have so many vastly different genetic mutations associated with the same behavior, such as autism or cerebellar ataxia or parkinsonism.
